# Using pens as an incentive for trial recruitment of older adults: An embedded randomised controlled trial

**DOI:** 10.12688/f1000research.18300.1

**Published:** 2019-03-21

**Authors:** Katie Whiteside, Lydia Flett, Alex Mitchell, Caroline Fairhurst, Sarah Cockayne, Sara Rodgers, David Torgerson, OTIS Study Group

**Affiliations:** 1 York Trials Unit, Department of Health Sciences, University of York, York, YO10 5DD, UK

**Keywords:** Randomised Controlled Trial, Embedded Trial, Recruitment, Incentive, Pen

## Abstract

**Background**: Meeting recruitment targets for randomised controlled trials is challenging.  This trial evaluated the effectiveness of including a pen within the trial invitation pack on the recruitment of older adults into a randomised controlled trial.

**Methods**: This trial was embedded within the Occupational Therapist Intervention Study, a falls-prevention randomised controlled trial.  Potential participants (n = 1862), who were posted an invitation pack from two General Practitioner practices, were randomised to either not receive a pen (n = 1295) or receive a pen (n = 648) with their invitation pack, using a 2:1 ratio.  The primary outcome was the likelihood of being randomised, and therefore fully recruited, to the host trial.  To be randomised to the host trial, participants had to: return a consent form and screening form; be eligible on their screening form; and return a baseline questionnaire and a monthly falls calendar.  Secondary outcomes were: the likelihood of returning (and time to return) a screening form; being eligible for the host trial; and remaining in the trial for at least 3 months.

**Results**: The likelihood of being randomised to the host trial did not differ between the pen group (4.5%) and no pen group (4.3%; odds ratio 1.04; 95% confidence interval: 0.65 to 1.67; *p* = 0.86).  There were marginal differences in secondary outcomes in favour of the pen group, particularly in screening form return rates, though these differences were not statistically significant.

**Conclusion**: Pens may not be an effective incentive for the recruitment of older adults into randomised controlled trials, though future trials are required.

**Registration:** ISRCTN22202133; SWAT 37.

## Introduction

Randomised controlled trials (RCTs) are vital in establishing the effectiveness of interventions. However, recruitment into RCTs remains a substantial challenge ^1^, with only around 55% of healthcare RCTs achieving their recruitment target and about 32% having to extend their recruitment period ^2, 3^. This can lead to underpowered trials that fail to find relevant group differences as statistically significant, as well as delayed results and increased costs due to recruitment extensions ^1^. Despite this, there is currently a lack of evidence to inform researchers of how recruitment into RCTs might best be improved ^1^, though this has been identified as a high priority to address ^4^. Therefore, it is crucial that potential strategies to improve recruitment are robustly evaluated by embedding RCTs evaluating such strategies into real ‘host’ RCTs ^5^.

One strategy to improve recruitment is the use of incentives. Based on the principle of reciprocity, receiving incentives is hypothesised to encourage individuals to respond to the positive behaviour in a positive way ^6, 7^. Both monetary and non-monetary incentives are frequently used by clinical trials units in the UK to support recruitment, despite a lack of evidence of their impact ^8^. Some evidence suggests that monetary incentives improve RCT recruitment rates ^1^; however, this strategy is expensive and ethically controversial ^9^. In contrast, non-monetary incentives, such as providing pens, are cheaper and more ethically sound ^10^.

The use of pens as an incentive is an especially appealing strategy for RCTs that utilise large-scale database recruitment. This method involves distributing invitation packs to individuals identified as potentially eligible for a trial from database searches (e.g. General Practitioner [GP] records) and is particularly suitable for recruiting participants with chronic conditions and for recruitment into RCTs that evaluate public health interventions ^11^. Database recruitment is minimally labour intensive, inexpensive, and associated with improved recruitment rates compared to opportunistic recruitment ^11^. Nevertheless, it would be valuable to explore how this strategy could be more efficient, as recruitment yield can still be low. This can especially be the case when recruiting older adults, a population faced with numerous barriers to trial participation, such as reduced mobility, a lack of trust and understanding of trials, and the belief that participation would be too burdensome ^12, 13^. For example, an RCT evaluating a podiatry intervention for falls prevention in older adults randomised just 2.7% of those approached via database recruitment into the trial ^14^.

The inclusion of pens within trial invitation packs may not only improve recruitment rates through encouraging reciprocal positive behaviour, but the convenience of a pen being readily available may prompt rapid completion and return of trial documentation ^15^. Despite this, no previous RCTs have evaluated the impact of distributing pens within invitation packs on recruitment into RCTs. However, a trial evaluating recruitment into a questionnaire survey reported that providing a study-branded pen within the invitation pack, to individuals who had previously not responded, improved response rates ^16^.

Some relevant trials have explored whether providing pens improves response rates to postal questionnaires, though these have yielded mixed findings. A previous trial found that sending a pen with a postal questionnaire to consultants did not improve response rates ^15^. However, other trials have reported that providing a study-branded pen or pencil was a cost-effective strategy which improved follow-up questionnaire response rates ^16, 17^. Similarly, a UK-based embedded RCT, evaluating an osteoporosis screening programme, found that including pens with postal questionnaires led to a marginal increase in response rates, a reduction in the number of reminders required, and a reduction in time to return the questionnaire ^10^. While the effects reported in this trial were all very small, the provision of pens was considered cost-effective due to their low cost. Given these promising results, it would be valuable for further embedded RCTs to evaluate whether these findings generalise to improvements in trial recruitment when a pen is included within the invitation pack.

In this paper we describe an embedded RCT (or ‘study within a trial’ [SWAT]) designed to evaluate the effectiveness of including a pen within the trial invitation pack on the recruitment of older adults, identified from GP database searches, into the Occupational Therapist Intervention Study (OTIS) ^18^. Specifically, this RCT evaluated the impact of providing pens on subsequent recruitment rates into the OTIS trial, as well as return rates of recruitment documentation, eligibility of respondents, and the retention of participants. This trial not only helped to address the lack of RCTs on the use of pens as an incentive for trial recruitment, but also explored how to further improve the efficiency of database recruitment, focusing specifically on older adults, who can be particularly challenging to recruit.

## Methods

### Design

This two-arm RCT was embedded within OTIS, which is a UK-based modified cohort RCT. The protocol for the OTIS trial has been published previously ^18^. In brief, the OTIS trial aimed to assess whether home environmental assessment and modification, led by an occupational therapist (OT), could reduce risk of falling among community dwelling, older adults at elevated risk of falling. Approval for the OTIS trial and this embedded trial was granted by the National Health Service West of Scotland Research Ethics Committee 3; the University of York, Department of Health Sciences Research Governance Committee; and the Health Research Authority. This embedded trial was registered with the ISRCTN registry as part of the host trial registration (ISRCTN22202133; date registered: 20 ^th^ June 2016) and was also registered with the Northern Ireland Hub for Trials Methodology Research SWAT Repository (SWAT 37; date registered: 20 ^th^ February 2016).

### Participant recruitment and intervention

One of the main recruitment methods for the OTIS trial was GP mail-outs. We embedded this trial in mail-outs from two UK-based GP practices. Within these mail-outs, men and women identified as potentially eligible (i.e. aged over 65 years and community dwelling) in database searches were posted a trial invitation pack. These packs included an invitation letter, a participant information sheet, consent form, screening form, and a pre-paid return envelope. Participants allocated to the intervention group of this embedded trial also received a York Trials Unit branded pen in their invitation pack. The control participants did not receive a pen in their invitation pack. Recipients of an invitation pack were asked to return a completed consent form and screening form if they were willing to take part in the OTIS trial.

To be eligible for the OTIS trial, participants had to be: over 65 years, community dwelling, currently able to walk 10 feet (with a walking aid if needed), willing and able to provide informed consent and to receive an OT home visit, and must not have had an OT assessment in the previous 12 months or be on the waiting list for one. Additionally, participants had to have one of the following risk factors for falling: have had at least one fall in the past 12 months; or report that they worry about falling at least some of the time. Participants who were eligible except for fulfilling a risk factor for falling were contacted again 4 to 6 months later for rescreening.

Eligible participants were then posted a baseline questionnaire to complete along with an Age UK falls prevention advice leaflet, and monthly falls calendars to return at the start of each month with details of any falls they had during the previous month (for up to 12 months after randomisation). Once participants had returned their completed baseline questionnaire and at least one falls calendar, they become eligible to be randomised into the OTIS trial to either receive an OT home visit or usual care.

Recruitment of embedded trial participants into the host trial commenced in May 2017 and follow-up for this embedded trial ended in May 2018.

### Sample size and randomisation

As is typical for an embedded trial, a formal sample size calculation was not carried out. The sample size was constrained by the number of invitation packs distributed via GP mail-outs during the time-period in which this embedded trial took place. Allocation to either the intervention ‘pen’ arm (to receive a York Trials Unit branded pen with the invitation pack) or the control ‘no pen’ arm (to receive the invitation pack with no pen) was achieved using block randomisation stratified by GP practice. We used a 2:1 allocation ratio, in favour of the no pen arm. Unique participant identification numbers for each invitation pack, prepared for the two GP practice mail-outs involved in this embedded trial, were randomised within three blocks. A single randomisation block, the size of the full mail-out, was used for the first GP practice mail-out and two blocks, of roughly equal size, were used for the second GP practice mail-out. Generation of the allocation sequence was undertaken by the OTIS trial statistician, who was not involved with production of the invitation packs, using Stata version 13 ^19^.

### Outcomes

The primary outcome was the proportion of embedded trial participants who were randomised into the OTIS main trial. Secondary outcomes were:

a) proportion of participants who returned a screening form;
b) time to return screening form;
c) proportion of participants who were initially 'pending' in terms of their eligibility on initial screening (i.e. fulfilled all eligibility criteria apart from a risk factor for falling);
d) proportion of participants who were eligible on initial screening;
e) proportion of participants who remained in the trial at three months post randomisation (defined as returning at least the first three months’ worth of falls calendars post-randomisation).

### Statistical analysis

Data were analysed on an intention-to-treat basis using two-sided tests at the 5% significance level. Categorical data were compared using logistic regression models and time to response data were analysed using a Cox proportional hazards model. All models adjusted for the GP site the invitation packs were mailed out from. Additionally, the logistic regression model used to analyse trial retention adjusted for the OTIS trial group allocation (usual care or intervention). The odds ratio (OR) or hazard ratio (HR) from each model associated with the pen embedded trial allocation is presented along with the corresponding 95% confidence interval (CI) and *p*-value. All analyses were conducted using Stata version 15 ^20^.

## Results

**Figure 1.**
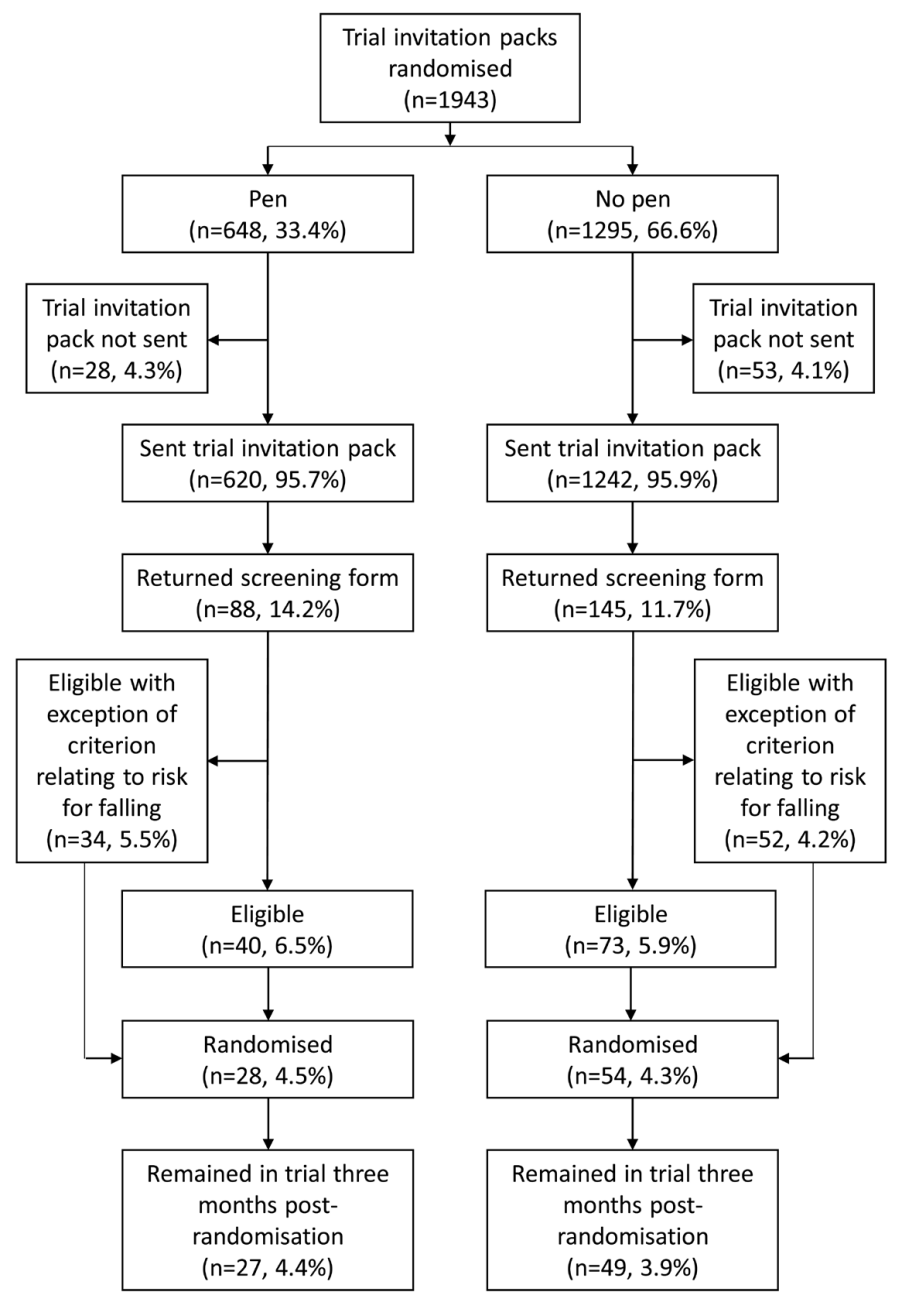
Flow diagram depicting the recruitment and retention of participants in this embedded trial.

We randomised 1943 participants into this embedded trial (648 to receive a pen with their invitation pack; 1295 to not receive a pen); however, 81 of these invitation packs were not sent out (pen arm = 28; no pen arm = 53) and were excluded from this analysis ( Figure 1). Therefore, we included 1862 participants in this analysis (pen arm = 620, 33.3%; no pen arm = 1242, 66.7%). Of these participants, 919 (49.4%) were posted an invitation pack from GP practice 1 and 943 (50.6%) were posted a pack from GP practice 2. Raw data are available on Open Science Framework ^21^.

### Randomisation rate

Of the 1862 embedded trial participants, 82 (4.4%) were randomised into the OTIS trial (pen: 28/620 [4.5%]; no pen: 54/1242 [4.3%]; difference of 0.17%; 95% CI of difference: -1.82% to 2.16%). The two groups did not significantly differ in their likelihood of being randomised into the OTIS trial (OR 1.04; 95% CI: 0.65 to 1.67; *p* = 0.86).

### Screening form return rate

In total, 233 (12.5%) of the 1862 embedded trial participants returned a screening form (pen: 88/620 [14.2%]; no pen: 145/1242 [11.7%]). The two groups did not significantly differ in their likelihood of returning a screening form (OR 1.25; 95% CI: 0.94 to 1.67; *p* = 0.12).

### Time to return screening form

For the 233 screening forms returned, the median time to return was 22 days (interquartile range [IQR]: 17 to 29) in the pen arm and 20 days (IQR: 17 to 28 days) in the no pen arm. There was no statistically significant difference in the time to respond between the two arms (HR 1.23; 95% CI: 0.94 to 1.60; *p* = 0.13). As the response rate was less than 50%, the median time to return a screening form could not be calculated from Kaplan-Meier survival estimates, so the 10 ^th^ percentile survival times were estimated instead. It took 26 days (95% CI: 24 to 39) in the pen arm, and 39 days (95% CI: 25 to 119) in the no pen arm, for 10% of the mailed screening forms to be returned.

### Pending eligibility rate

Of the 1862 embedded trial participants, 86 (4.6%) were initially ‘pending’ in their eligibility for the OTIS trial, whereby they met all eligibility criteria apart from a risk factor for falling (pen: 34/620 [5.5%]; no pen: 52/1242 [4.2%]). The two groups did not significantly differ in their likelihood of having an eligibility status of ‘pending’ on initial screening (OR 1.33; 95% CI: 0.85 to 2.07; *p* = 0.21).

### Eligibility rate

In total, 113 (6.1%) of the 1862 embedded trial participants were eligible for the OTIS trial on their initial screening form (pen: 40/620 [6.5%]; no pen: 73/1242 [5.9%]). The two groups did not significantly differ in their likelihood of being fully eligible on initial screening (OR 1.11; 95% CI: 0.74 to 1.65; *p* = 0.62).

### Retention rate

Of the 1862 embedded trial participants, 76 (4.1%) remained in the OTIS trial 3 months post-randomisation (pen: 27/620 [4.4%]; no pen: 49/1242 [3.9%]). There was no statistically significant difference in the number of embedded trial participants remaining in the OTIS trial 3 months post-randomisation between the two groups (pen: 27/28 [96.4%]; no pen: 49/54 [90.7%]; OR 2.63; 95% CI: 0.29 to 24.1; *p* = 0.39). This analysis adjusted for OTIS trial group allocation and therefore the sample size (n = 82) reflected the number of embedded trial participants randomised into the OTIS trial.

## Discussion

This embedded RCT evaluated the effectiveness of including a non-monetary incentive, in the form of a York Trials Unit branded pen, within invitation packs mailed out from GP practices on the recruitment of older adults into the OTIS trial. The absolute difference in the percentage of embedded trial participants randomised to the OTIS trial was 0.17% (4.5% in the pen arm, 4.3% in the no pen arm) and was not statistically significant, which suggests that providing a pen within trial invitation packs was not an effective incentive to improve recruitment of older adults into the host RCT.

Whether providing pens as a recruitment incentive is cost-effective remains uncertain. Based on the randomisation rate of 4.3% (or 43 per 1,000) achieved in this embedded trial using standard invitation packs, and given that the printing, packaging, and postage costs for each standard pack was £2.53, it costs £2,530 to send 1,000 standard packs to recruit 43 participants into the host trial, or £58.84 per participant. The pens cost £0.32 each, so it costs an additional £320 per 1,000 packs distributed with a pen. For this price, approximately five participants could be recruited using standard packs; therefore, including a pen would need to increase the percentage of eligible participants randomised by 0.5% (or 5 per 1,000) to be cost-effective. If the point estimate reported here (0.17%) is the true difference, providing pens would not be cost-effective. However, if the upper 95% confidence limit of the difference (2.16%) is the true difference, providing pens would likely be cost-effective. Consequently, additional trials are needed to evaluate this recruitment strategy. Furthermore, as the cost of pens could be reduced if a non-branded style were used, further trials could additionally evaluate the effectiveness of the branding.

Within this embedded RCT, the provision of pens was not associated with a significant difference in any of the secondary outcomes, though all results favoured the pen arm. Providing a pen in the invitation pack resulted in a small increase in screening form return rates (absolute difference of 2.5%). There were also trends for those who received a pen to return their screening form more quickly and to be more likely to remain in the OTIS trial for at least 3 months after being randomised (96.4% vs. 90.7%). These results suggest that including a pen in trial invitation packs may marginally boost the return of trial recruitment documentation among older adults, a population that can be particularly difficult to recruit ^12, 13^, and may have benefits on trial retention. While improvements in screening form response rates did not translate to improvements in randomisation rates in this trial, it is possible that it may do in other trials, particularly those with broader eligibility criteria. This further highlights the need for future trials to evaluate pens as a recruitment incentive.

This embedded RCT adds to the limited and mixed literature on the provision of pens on response rates to trial documentation, with some previous trials showing an effect ^10, 16, 17^ and others showing no effect ^15^. While previous trials have considered the impact of providing pens on questionnaire return rates, this trial was the first to evaluate the inclusion of a pen within the trial invitation pack on RCT recruitment.

This embedded trial was limited by only involving two GP practice mail-outs and focusing on older adults. It would be beneficial for future embedded trials, within large-scale RCTs utilising database recruitment, to involve a greater number of mail-outs to further evaluate this question. Further research should also explore the impact of providing pens on the recruitment of different participant populations (e.g. different age groups). Meta-analysis could then be used to explore the effectiveness of including pens within trial invitation packs and whether this varies depending on participant demographics.

## Conclusions

Providing a pen within trial invitation packs had marginally beneficial effects on screening forms return rates and retention within this embedded trial, though did not improve randomisation rates of older adults into the host RCT. Further embedded trials are necessary to evaluate whether providing pens in invitation packs is a cost-effective incentive for trial recruitment.

## Data availability

### Underlying data

Open Science Framework: Underlying data and CONSORT checklist for using pens as an incentive for trial recruitment of older adults: An embedded randomised controlled trial. https://doi.org/10.17605/OSF.IO/6FMGC ^21^. This project contains the following underlying data files:

- Dataset1_OTIS_pensubstudy_F1000_data.csv (raw data in CSV format)
- Dataset1_OTIS_pensubstudy_F1000_data.sav (raw data in SAV format)
- Dataset1_OTIS_pensubstudy_F1000_variable_key.csv (definition or abbreviations in dataset)

### Reporting guidelines

Open Science Framework: CONSORT checklist for “Using pens as an incentive for trial recruitment of older adults: An embedded randomised controlled trial”. https://doi.org/10.17605/OSF.IO/6FMGC ^21^

Data are available under the terms of the Creative Commons Zero "No rights reserved" data waiver (CC0 1.0 Public domain dedication).
