# Supplementary material for: Using pens as an incentive for trial recruitment of older adults: An embedded randomised controlled trial
Source: F1000Res. 2019 Mar 21;8:315. [Version 1] doi: 10.12688/f1000research.18300.1 (PMC6446499; doi:10.12688/f1000research.18300.1)
Supplement: Peer review history

# Peer review: Reviewer response for version 1

**DOI:** 10.5256/f1000research.20018.r46144

**Published:** 2019-04-01

**Authors:** Nicola Mills

**Affiliations:** 1 Population Health Sciences, Bristol Medical School, University of Bristol, Bristol, UK

This article reports findings from a randomised study within a host RCT assessing pens within trial invite packs as an incentive for recruitment. Recruitment to RCTs is often problematic and there is little evidence on effective methods to optimise recruitment, so research in this field is worthy. A Study within a Trial (SWAT), as undertaken by this study group, is a potentially effective and efficient way of generating robust evidence to redress the gap. The work is clearly presented, drawing upon evidence from key literature, and the methodology appears sound.

 I’m not wholly convinced by the authors' conclusion that more trials are needed given that this was a study with robust methodology undertaken in a population who ‘may’ be less mobile and therefore appreciative of a pen to hand, and given the existing literature already around pens as an incentive to participate in research. I would like to have seen a wider discussion on the use of incentives and initiatives more generally to optimise recruitment, with consideration of the reason for their findings and direction for future research. Overall though, a focus on strategies to optimise recruitment to RCTs to build on the evidence base should be encouraged and I therefore recommend this article be indexed.

I have read this submission. I believe that I have an appropriate level of expertise to confirm that it is of an acceptable scientific standard.

## References

[1] HindD: Referee Report For: Using pens as an incentive for trial recruitment of older adults: An embedded randomised controlled trial [version 1]. *F1000Research*.2019;8(315) : Reference source 10.12688/f1000research.18300.1PMC644649930984388

# Peer review: Reviewer response for version 1

**DOI:** 10.5256/f1000research.20018.r46146

**Published:** 2019-03-27

**Authors:** Daniel Hind

**Affiliations:** 1 School of Health and Related Research (ScHARR), University of Sheffield, Sheffield, UK

This article reports the findings of a study within a trial (SWaT), a methodological design currently promoted by at least one major funder within the UK. Many SWaTs, including this one, are aimed at improving participant recruitment to RCTs, the principal source of study failure.

 The study is well conducted and, in almost all regards, suitable to be indexed as is. I have only one observation, which regards the conclusion that more trials are warranted. It is not obvious to this reader that the background or results support this conclusion.

 In the background, the authors cite, as a warrant for this trial, Clark *et al.*'s paper (Clark *et al.*, 2001 ^1^) in which a pen did not improve the response rate of a survey. In White *et al.*’s study (White *et al.*, 2005 ^2^) the opposite was the case. In this study, the inclusion of a pen did not increase participant recruitment. 'The best of three' - the pen loses.

 So, if we're going to do this on a purely empirical basis, it would be useful to know at what point we will decide that the pen is ineffective and White's success can be put down to the play of chance. What are the 'stop-go' criteria?

 On the other hand, if we look at this more mechanistically, we could ask why an incentive which is insufficient to motivate people in this population group would be any more likely to motivate people in other population groups. Is there some plausible link between returning your form more quickly and consenting to study entry? It's not obvious to me that there is.

 Otherwise, the report is a useful addition to the growing evidence for what works in the conduct of trials and I recommend it to be indexed.

I have read this submission. I believe that I have an appropriate level of expertise to confirm that it is of an acceptable scientific standard.

## References

[1] ClarkTJKhanKSGuptaJK: Provision of pen along with questionnaire does not increase the response rate to a postal survey: a randomised controlled trial. *J Epidemiol Community Health*.2001;55(8) :595-6 1144901910.1136/jech.55.8.595PMC1731952

[2] WhiteECarneyPAKolarAS: Increasing response to mailed questionnaires by including a pencil/pen. *Am J Epidemiol*.2005;162(3) : 10.1093/aje/kwi194 261-6 10.1093/aje/kwi194 15972931
